# Removal of Misplaced Left Ventricular Single Lead Pacemaker in a Patient Presenting with Recurrent Transient Ischemic Attacks

**DOI:** 10.51894/001c.6068

**Published:** 2017-08-24

**Authors:** Andrew Hinojos, Karl Ilg

**Affiliations:** 1 1 Genesys Regional Medical Center Internal Medicine Resident, PGY 3, Grand Blanc, MI; 2 Genesys Regional Medical Center Cardiology Core Faculty, Grand Blanc, MI

**Keywords:** atrioventricular node ablation, transient ischemic attack, cardiac pacemaker

## Abstract

Over 200,000 cardiac electronic implantable devices are annually placed in individuals living within the United States. Complications from this procedure can range up to 12%. Inadvertent lead placement into the left ventricle is a rare but recognized complication of implantable cardiac electronic devices. This is a retrospective case report of a female patient in her late 70’s who underwent atrioventricular node ablation and misplacement of single lead pacemaker, subsequently presenting with recurrent transient ischemic attacks one month later. Initial electrocardiogram and chest X-ray demonstrated misplacement of her pacemaker in the left ventricle. Medical therapy was attempted, however, patient subsequently underwent extraction via aortotomy with implantation of epicardial pacemaker. Inadvertent placement of implantable electronic cardiac devices is a rare but well recognized complication. A post-procedure electrocardiogram and chest X-ray should be routinely performed to confirm appropriate lead placement. Procedures to manage this complication are evolving with novel device therapies specifically designed for percutaneous lead extraction.

## INTRODUCTION

It is estimated that over 2.4 million people live with cardiac electronic implantable devices (CEID), with complication rates ranging up to 12%.^1,2^ The most common CEID-related complications include lead dislodgement, pocket hematoma/bleeding, pneumothorax, and infection.^1,3^ Inadvertent lead placement into the left ventricle (LV) is a rare but recognized complication of CEID use.^2,4–15^ This paper presents a case report of a patient who underwent atrioventricular node (AVN) ablation with subsequent misplacement of her single lead pacemaker.

### Case Report

A female in her late 70’s with a history of mitral valve repair suffered from long-standing symptomatic atrial fibrillation. After four electrophysiology studies with pulmonary vein isolation, repeat left atrium ablation, and flutter line ablation at the authors’ community hospital, the patient ultimately elected to receive an AVN ablation and implantation of a single lead pacemaker. She opted to go to an outside hospital for this procedure and her AVN ablation was complicated due to her difficult anatomy. According to available documentation, her coronary sinus had an early truncation into three possible venous branches, making it difficult to access the LV for pacing. The procedure was aborted and she instead had a single lead pacemaker implanted by the operating physician. The patient did well post-operatively and she was discharged home.

**Figure 1:**
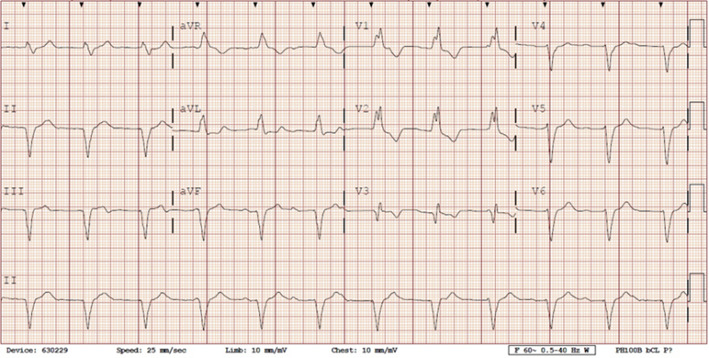
12-lead Electrocardiogram Consistent with Right Bundle Branch Block (RBBB)

**Figure 2:**
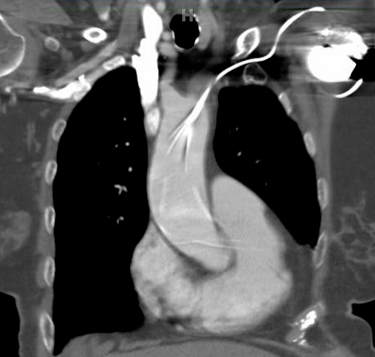
CT Chest Demonstrating Misplaced Single Lead Pacemaker

She presented to the authors’ hospital one month later with an episode of left-sided weakness, slurred speech, and the patient was diagnosed with a transient ischemic attack (TIA). Cardiology and neurology consults were obtained. She had been receiving warfarin for her atrial fibrillation and her international normalized ration (INR) on admission was at a therapeutic level. The authors could not exclude transient subtherapeutic INR levels leading to clot formation and thromboembolic phenomenon from her atrial fibrillation. However, her electrocardiogram (ECG) demonstrated an abnormal finding that provided an important clue for the etiology of her presenting symptoms. Her ECG demonstrated a ventricular paced rhythm with a right bundle branch block pattern (Figure 1). A chest x-ray was performed that demonstrated misplacement of the single lead pacemaker with an atypical posterior curvature path. A computerized tomography (CT) scan was performed which further demonstrated her misplaced single-lead pacemaker traversing the aortic valve into the LV (Figure 2).

As she was already on Coumadin for valvular atrial fibrillation, her target INR level was increased to a goal of 3.0-3.5 and she was discharged home. Four months later, she presented to the authors’ hospital with a second TIA.

Due to her recurrent TIAs from the misplaced lead, the authors decided to pursue surgical retrieval. She underwent pre-operative catheterization to assess any burden of coronary artery disease and a peri-operative trans-esophageal echocardiogram. The patient subsequently had an extraction of her LV pacemaker wire via aortotomy and implantation of an epicardial pacemaker. To date, the patient has been doing well without any further sequelae.

## DISCUSSION

Misplacement of CEIDs in the LV is a rare but recognized procedural complication. The most common etiologies occur when a catheter traverses through the interatrial septum, patent foramen ovale or atrial septal defect.^2–8^ Less commonly, leads may perforate the interventricular or the atrioventricular septum and deploy in the LV.^4,8,9^ Implantation through an intra-arterial approach that crosses the aortic valve is even more rare.^3,9–13^ Our case described a misplaced LV pacemaker lead via a subclavian artery insertion traversing the aortic valve and implanting into the LV.

Complications from a misplaced CEID can result in both significant neurologic and vascular sequelae. Previous reports have indicated a thromboembolic complication rate has ranged from 37 to 67%.^2–4,6^ Cerebral thromboembolic complications are the most serious, ranging from mental confusion to cerebrovascular accident with permanent neurologic deficits.^4,5^ Vascular complications most often occur on access of the subclavian artery and extraction of the misplaced CEID.^5^ Potential complications from the procedure include bleeding, hematoma formation, loss of brachial and/or radial pulse, and arterial thrombus formation.^5^ Less commonly, misplaced CEID involving the left side of the heart can result in damage to mitral and aortic valves or trauma to the coronary arteries leading to myocardial ischemia and/or infarction.^4,6,10^

Both ECG and chest x-rays can provide clues for potential misplacement of ventricular pacemakers. Proper placement in the apex of the right ventricle will demonstrate a left bundle branch morphology (LBBB) on ECG. A right bundle branch block (RBBB) pattern should raise suspicion for left ventricular stimulation due to lead malposition. Although uncommon, a RBBB can result if the pacing electrode enters the coronary sinus and into posterior interventricular vein, inserts into the interventricular septum, or with normal pacing in a dilated right ventricle.^5–7,10^ Notably, 8 to 13% of patients will still have a RBBB morphology on ECG with normal right ventricular pacing.^9^ While a post procedure ECG should be performed for confirmation of proper lead placement of every CEID, this test cannot entirely confirm lead placement.

On chest x-ray, both anterior-posterior (AP) and lateral views are preferable for confirmation of proper pacemaker lead placement. A lateral view film is very important to evaluate positioning since leads placed in the right ventricle have a lateral and forward direction on imaging while placement in coronary sinus or LV have a backward direction on lateral chest x-rays. An abnormal posterior position of the catheter tip on a lateral chest x-ray film should raise suspicion of improper lead placement.^3,5,6,9,12^ On AP films, a misplaced lead in the LV may present as an abnormally high “takeoff” of the ventricular lead.^6^

A chest x-ray alone cannot confirm malposition of a misplaced lead, as a posterior catheter position can be a result of an electrode in the coronary sinus.^12^ Thus, AP and lateral films can be used as a tool to confirm proper placement of CEIDs post-implantation. However, further imaging should be pursued if there is any suspicion of lead misplacement.

Confirmation of venous access can be difficult, such as in cases of congenital heart disease where arterial blood may appear darker than expected. Thus, there are maneuvers that the operator can perform to help ensure proper placement of the lead in the venous system. During implantation, the guidewire should always be advanced to the inferior vena cava or the pulmonary trunk to ensure venous access. In addition to the standard anterolateral views under fluoroscopy, left anterior oblique or right anterior oblique views should also be performed.^2^ After implantation, a post-operative ECG and pair of frontal and lateral x-rays should be routinely performed to confirm proper pacemaker placement.^10^

Finally, echocardiography is the test of choice for confirming position of a misplaced CEID. Echocardiograms can directly visualize implantation and enable the provider to follow the course of the misplaced CEID. Echocardiography can also identify a thrombus that may be attached to the misplaced pacemaker, or pericardial effusion, which may indicate myocardial perforation.^2,5,7,8^

Lead removal, however, presents a risk of thromboembolic event, and thrombus may not always be identified on echocardiography.^2^ Among those individuals suffering thromboembolic complications, 10% to 20% have had a thrombus identified on echocardiogram prior to extraction.^2,4^ Thus, preoperative echocardiography cannot be used to reliably identify presence of thrombus but can be used to confirm lead misplacement.

Treatment of a misplaced CEID is comprised of two options: anticoagulant therapy or surgical removal. Decisions concerning which approach is most suitable are based on patient’s symptoms and surgical risks.^6^ General recommendations for inadvertent lead placement in an asymptomatic patient with higher surgical risks are to initiate anticoagulation using Coumadin/warfarin with a goal INR of 2.5 to 3.5.^2–6^ Antiplatelet therapy has been shown to pose a high incidence of thromboembolic events and is not recommended as primary therapy.^2,4^

Surgical removal is often tried in patients that experience thromboembolic complications despite anticoagulation or for patients with prior scheduled cardiac surgery.^2–4^ More recently percutaneous lead extraction has been performed successfully, although there is a higher risk of dislodging thrombi or fibrotic adhesions as time to extraction increases.^2,6^

Percutaneous extraction can be performed with simple manual traction, traction devices, sheaths that use laser or electrosurgical energy, or sheaths with rotating threaded tips.^2,6^ Traction devices include specialized locking stylets, snares, sutures, or grasping devices used to engage/entrap and remove the lead or lead fragments. Sheaths require manual advancement over the lead and rely on the mechanical properties of the sheath to disrupt fibrotic attachments. Laser sheaths use fiber optics to transmit laser light while electrosurgical sheaths use radiofrequency energy emitted between two electrodes at the sheath tip to disrupt the fibrotic attachments. Major complications from device extraction is approximately 1.4%.^15^ Complications rates are higher with longer implant durations and use of laser extraction technique.^15^

In 2011, Rodriquez et al. described favorable outcomes from both open surgical and percutaneous extraction techniques.^2^ The case series published by this group suggested that simple manual traction can be attempted in patients with implantation of less than six months. Older pacemaker leads often have additional fibrosis and may require laser energy for removal.^4^

Although case reports of successful percutaneous removal of chronic leads have been reported, this practice is not generally recommended due to the risk of creating emboli or dislodging thrombi into systemic circulation.^2,7,13^ With developing technologies, percutaneous lead extraction may be reserved for special cases where surgical extraction may be complicated due to a candidate with higher surgical risks.

## CONCLUSIONS

Misplacement of CEIDs is a rare but recognized complication. This case study presented a patient with a misplaced single lead pacemaker via the subclavian artery implanting into the LV. As the patient presented with recurrent TIAs despite medical therapy, the device was removed surgically via aortotomy. Patients with a CEID who present with neurologic symptoms should be screened for proper lead placement with an ECG and/or set of chest x-rays. Although this patient underwent an open surgical removal, novel technologies designed for percutaneous device extraction of misplaced CEID are continuing to improve.

### Conflict of Interest

The authors declare no conflict of interest.
